# Ramipril-associated cholestasis in the setting of recurrent drug-induced liver injury 

**Published:** 2017

**Authors:** David Forner, Tasha Kulai, Thomas Arnason, Steven E. Gruchy, Magnus MacLeod

**Affiliations:** 1 *Department of Medicine, Dalhousie University, Halifax, Nova Scotia, Canada*; 2 *Division of Digestive Care and Endoscopy, Dalhousie University, Halifax, Nova Scotia*; 3 *Department of Pathology, Dalhousie University, Halifax, Nova Scotia, Canada*

**Keywords:** Drug-induced liver injury (DILI), Ramipril, Angiotensin-converting enzyme inhibitors, Liver, Cholestasis, Methimazole

## Abstract

**Aim:**

Angiotensin-converting enzyme inhibitors (ACEIs) are commonly used to treat hypertension. Although generally well tolerated, the adverse effects of ACEIs include hypotension, cough, acute kidney injury and hyperkalemia. Rare reports of ACEI-induced hepatotoxicity have been described, most notably a cholestatic pattern of injury related to captopril.

A 67-year-old male presented to the emergency department with a three-week history of jaundice, pruritis and weakness. Eight weeks before, he began taking ramipril and clopidogrel. His past medical history was significant for previous acute cholestatic liver injury approximately 20 years earlier, which was attributed to methimazole. Abnormal blood work demonstrated aspartate aminotransferase (AST) 47 U/L, alanine aminotransferase (ALT) 46 U/L, total bilirubin 230 µmol/L, direct bilirubin 176 µmol/L, and alkaline phosphatase (ALP) 470 U/L. Abdominal ultrasound and magnetic resonance cholangiopancreatography showed no bile duct obstruction. Further work-up was negative for infectious, autoimmune, or other causes. Percutaneous liver biopsy showed marked cholestasis. With discontinuation of ramipril, the patient demonstrated prolonged cholestasis with partial biochemical improvement and was discharged after six weeks in hospital.

This case represents the first described cross reactivity between ramipril and methimazole, illustrating the complex and poorly understood nature of DILI. Despite the relatively few instances of ACEI-induced liver hepatotoxicity, consideration should be given to discontinuation of ramipril in situations of unknown liver damage.

## Introduction

 Angiotensin-converting enzyme inhibitors (ACEIs) are commonly used to treat hypertension. By inhibiting peptidyl dipeptidase and blocking the conversion of angiotensin I to angiotensin II, ACEIs block the renin angiotensin aldosterone system and inhibit bradykinin inactivation, resulting in an overall hypotensive effect. Ramipril is an oral prodrug that is de-esterified to ramiprilat through first pass effect by the liver, and as such is a long-acting member of the ACEI class. The prodrug and its metabolites are eliminated through combined kidney and biliary excretion. In fact, the kidney, with the exception of fosinopril and moexipril, eliminates all ACEIs (1). 

Although generally well tolerated, the adverse effects of ACEIs include hypotension, cough, acute kidney injury and hyperkalemia (1). Rare reports of ACEI-induced hepatotoxicity have been described, most notably a cholestatic pattern of injury related to captopril (2). 

## Case Report

 A 67-year-old male presented to the emergency department with a three-week history of jaundice, pruritis and weakness. Eight weeks before, he began taking ramipril and clopidogrel after sustaining an inferior wall ST-elevation myocardial infarct. Changes to his original home medications also included increased dosing of bisoprolol and atorvastatin. He was first seen by outpatient internal medicine with the same symptoms two weeks before presenting to the emergency department and atorvastatin was discontinued; however, he continued to worsen clinically and biochemically. His past medical history was significant for previous acute cholestatic liver injury approximately 20 years earlier, which was attributed to methimazole after a negative work-up for causes of liver disease. 

**Figure 1:**
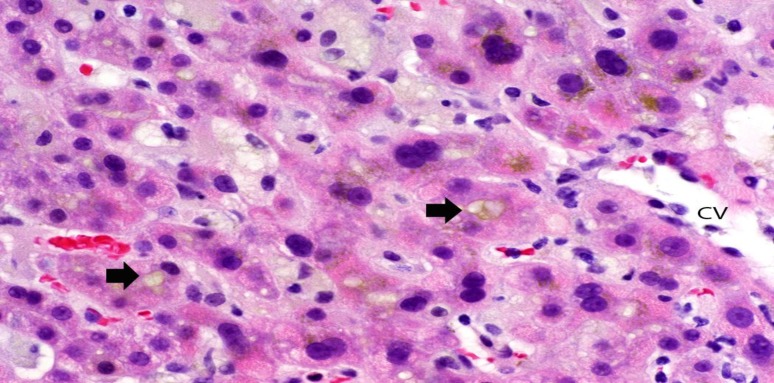
A liver biopsy shows canalicular cholestasis (at arrows) as well as cholestasis within hepatocytes in zone 3, near the central vein (CV). Some hepatocytes have edematous, vacuolated cytoplasm, which often occurs with cholestasis (hematoxylin and eosin, original magnification x 400

Physical examination revealed jaundice, but was otherwise unremarkable. Abnormal blood work demonstrated aspartate aminotransferase (AST) 47 U/L, alanine aminotransferase (ALT) 46 U/L, total bilirubin 230 µmol/L, direct bilirubin 176 µmol/L, alkaline phosphatase (ALP) 470 U/L, INR 1.4 and albumin 29 g/L. Abdominal ultrasound with Doppler and magnetic resonance cholangiopancreatography showed no bile duct obstruction. Further work-up was negative for infectious (Hepatitis A IgM, Hepatitis B surface antigen, Hepatitis C antibody screen, human immunodeficiency virus antibody and antigen screens, parvovirus B19 IgM, acute mononucleosis screen and Q Fever serology), autoimmune (anti-tissue transglutaminase IgA, immunoglobulins, anti-nuclear antibody screen, anti-mitochondrial antibody, anti-smooth muscle antibody and IgG-4 subclass serologies), and other (hereditary hemochromatosis) causes. Percutaneous liver biopsy showed marked cholestasis (Figure 1). There was minimal portal-based inflammation and no interface or lobular hepatitis. Features of large duct obstruction (portal edema, ductular reaction) were not apparent. There was no fibrosis on review of trichrome stains. The pathology was felt to be compatible with medication-induced cholestasis. His previous pathology report from 20 years earlier noted a similar histologic pattern of bland cholestasis. 

His hospital stay was complicated by urinary retention, epididymitis and poor oral intake with subsequent orthostatic hypotension. With discontinuation of ramipril, the patient demonstrated prolonged cholestasis with partial biochemical improvement and was discharged after six weeks in hospital. One month after discharge, his total bilirubin had decreased to 35.5 µmol/L and ALP to 269 U/L

## Discussion

Idiosyncratic drug-induced liver injury (DILI) is rare, occurring at an incidence of 19 cases per 100,000 people per year according to a recent prospective population-based study from Iceland (3). Both genetics and environment are hypothesized to play a role in the development of DILI. For example, the HLA allele DRB1*1501 has been found to be associated with amoxicillin-clavulin derived cholestastic liver injury (4). Similarly, HLA DRB*0701 is associated with DILI due to ximelagatran (5). Several other risk factors have also been identified for the development of DILI, including age (above 55 years), gender (female), drug dose and alcohol use (6). Diagnosis can be challenging as the clinical presentation of DILI varies widely and confounding medications are often present. Here, causality assessment with the Council for International Organizations of Medical Sciences scale was 7 (probable) (7). 

This case report is the fifth known case of ramipril-induced hepatotoxicity (2, 8). However, there have been over 50 reports of drug-induced hepatotoxicity associated with other ACEIs, the most common being captopril (8, 9). Cases varied in their symptomatic presentation, from incidental findings on blood work to jaundice and severe pruritus. Cholestasis appears to be the most common liver injury pattern in ACEI-induced hepatotoxicity, and this pattern has been evident in two previous cases of ramipril-induced liver injury with biopsies revealing cholestasis and bile duct necrosis (2). Following discontinuation of the offending agent, biochemical markers returned to near baseline at 6 and 14 months, respectively. In the case presented here, biopsy revealed bland cholestasis with no ductular changes and both alkaline phosphatase and bilirubin began declining promptly after discontinuation of ramipril. However, at one month post discharge, the patient’s biochemical markers had not yet returned to baseline.

The remaining two previously reported cases of ramipril-induced liver injury manifested as unclassifiable and mixed. The unclassifiable case exhibited only mild increases in liver enzymes and a biopsy was not performed (2). The mixed pattern presented with markedly elevated ALT and ALP, with only minimal increases in bilirubin. Negative investigations prompted biopsy, revealing parenchymal inflammation. Re-institution resulted in an unintended positive rechallenge, confirming the diagnosis of ramipril-induced livery injury (8). The period of time from initiation of ramipril to presentation varied between cases, ranging from five weeks to 10 months. The current case is within this window, presenting eight weeks after beginning ramipril. 

Three key mechanisms have been previously suggested as the cause of ramipril-induced hepatotoxicity, including metabolic interaction, hypersensitivity, and bradykinin-mediated effects, although no model currently exists to confirm these hypotheses. In previous ramipiril-induced liver injury reports, both peripheral eosinophilia and biopsy proven eosinophil infiltration were seen, supporting the mechanism of hypersensitivity (2). However, no eosinophils were seen on biopsy in the current patient, nor was peripheral eosinophilia present. The majority of ACEIs differ only slightly in their chemical structure, supporting a possible metabolic interaction that is relatively conserved across the three structural classes by which ACEIs are categorized (10). Finally, similarly to other ACEIs, ramipril causes an increase in bradykinin through a reduction in bradykinin inactivation, resulting in an increase in prostaglandin synthesis. In turn, specific prostaglandins have been shown to cause decreased gallbladder contraction and bile stasis in humans (11). Similarly, the mechanism of methimazole-induced hepatotoxicity is unknown, although immune-mediated toxicity and reactive metabolite formation are suspected to be involved (12).

Recurrent DILI is a rare and poorly understood phenomenon, occurring in 1.21% of patients who experience it (13). Immune cross-sensitization by the two implicated drugs is a potential mechanism. Additionally, shared structural or functional aspects of the medications themselves and genetic predisposition, such as the presence of particular major histocompatibility complex regions, may play a role. Recurrent hepatotoxicity of the same phenotype (cholestasis in this case) is typical for patients with a second episode of drug-induced liver injury, regardless of the causative medication, for unclear reasons (13). 

Although uncommon, the case of ramipril-induced livery injury described here illustrates the need for thoughtful consideration of all medications as potential causes of DILI. This case represents the first described cross reactivity between ramipril and methimazole, illustrating the complex and poorly understood nature of DILI. Despite the relatively few instances of ACEI-induced liver hepatotoxicity, consideration should be given to discontinuation of ramipril in situations of unknown liver damage.

This case represents a rare report of ACEI-induced cholestatic hepatotoxicity in a 67-year-old male with previous DILI, making this the first case of cross reactivity between ramipril and methimazole. The mechanism of hepatotoxicity is poorly understood in both medications. The cholestasis seen in ramipril-induced liver injury can be prolonged. In patients presenting with hepatotoxicity of an unclear etiology, a careful review of medications is warranted and discontinuation of potential offending drugs should be considered.

## Conflict of interest

The authors declare that they have no conflict of interest.
